# Lived experiences: a focus group pilot study within the MentALLY project of mental healthcare among European users

**DOI:** 10.1186/s12913-020-05454-5

**Published:** 2020-07-01

**Authors:** Malin Axelsson, Viktor Schønning, Claudi Bockting, Ann Buysse, Mattias Desmet, Alexis Dewaele, Theodoros Giovazolias, Dewi Hannon, Konstantinos Kafetsios, Reitske Meganck, Spyridoula Ntani, Kris Rutten, Sofia Triliva, Laura Van Beveren, Joke Vandamme, Simon Øverland, Gunnel Hensing

**Affiliations:** 1 Department of Care Science, Faculty of Health and Society, Malmö University, Jan Waldenströms gata 25 – F416, SE-205 06 Malmö, Sweden; 2 Department of Public Health and Community Medicine, Institute of Medicine, The Sahlgrenska Academy at University of Gothenburg, Gothenburg, Sweden; 3 Department of Health Promotion, Division of Mental and Physical Health, Norwegian Institute of Public Health, Bergen, Norway; 4 Amsterdam UMC, Department of Psychiatry (AMC) and Institute for Advanced Studies, University of Amsterdam, Amsterdam, the Netherlands; 5 Department of Experimental Clinical and Health Psychology, Faculty of Psychology and Educational Sciences, Ghent University, Ghent, Belgium; 6 Department of Psychoanalysis and Clinical Consulting, Faculty of Psychology and Educational Sciences, Ghent University, Ghent, Belgium; 7 Department of Psychology, University of Crete, Rethymno, Crete Greece; 8 Aristotle University of Thessaloniki, Thessaloniki, Greece; 9 Department of Educational Studies, Faculty of Psychology and Educational Sciences, Ghent University, Ghent, Belgium; 10 Division of Mental and Physical Health, Norwegian Institute of Public Health & Department of Psychosocial Science, University of Bergen, Bergen, Norway

**Keywords:** Access, Clients, Collaboration, Diagnosis, Lived experiences, Mental health literacy, Referral, Patients, Service-users, Stigma, Treatment

## Abstract

**Background:**

Mental healthcare is an important component in societies’ response to mental health problems. Although the World Health Organization highlights availability, accessibility, acceptability and quality of healthcare as important cornerstones, many Europeans lack access to mental healthcare of high quality. Qualitative studies exploring mental healthcare from the perspective of people with lived experiences would add to previous research and knowledge by enabling in-depth understanding of mental healthcare users, which may be of significance for the development of mental healthcare. Therefore, the aim of the current study was to describe experiences of mental healthcare among adult Europeans with mental health problems.

**Method:**

In total, 50 participants with experiences of various mental health problems were recruited for separate focus group interviews in each country. They had experiences from both the private and public sectors, and with in- and outpatient mental healthcare. The focus group interviews (*N* = 7) were audio recorded, transcribed verbatim and analysed through thematic analysis. The analysis yielded five themes and 13 subthemes.

**Results:**

The theme *Seeking and trying to find help* contained three subthemes describing personal thresholds for seeking professional help, not knowing where to get help, and the importance of receiving help promptly. The theme *Awaiting assessment and treatment* contained two subthemes including feelings of being prioritized or not and feelings of being abandoned during the often-lengthy referral process. The theme *Treatment: a plan with individual parts* contained three subthemes consisting of demands for tailored treatment plans in combination with medications and human resources and agreement on treatment. The theme *Continuous and respectful care relationship* contained two subthemes describing the importance of continuous care relationships characterised by empathy and respect. The theme *Suggestions for improvements* contained three subthemes highlighting an urge to facilitate care contacts and to increase awareness of mental health problems and a wish to be seen as an individual with potential.

**Conclusion:**

Facilitating contacts with mental healthcare, a steady contact during the referral process, tailored treatment and empathy and respect are important aspects in efforts to improve mental healthcare. Recommendations included development of collaborative practices between stakeholders in order to increase general societal awareness of mental health problems.

## Background

Mental health problems are among the dominant causes of non-fatal health loss in Europe [1] affecting 17.3% (*n* = 84 million) of the European population [2]. The high prevalence of mental health problems and associated needs for mental healthcare pose a significant challenge for politicians and healthcare providers all over Europe [2, 3]. Still, a large proportion of Europeans lack access to high quality mental healthcare. Delayed or ineffective treatment of mental health problems has negative consequences for the individual but also for society, as it affects work participation and contributes to increased sickness absence [3].

The World Health Organization (WHO) has developed a framework, Availability, Accessibility, Acceptability and Quality of healthcare (AAAQ), as an analytic tool to clarify how the right to health [4], as stated in the United Nations (UN) declaration of universal human rights §25 [5], can be understood in terms of provision of healthcare (Table 1). The possibility to provide healthcare of high AAAQ standards is related to the overall healthcare system in each country, which in turn depends both on the economic situation and on political decisions [4]. The individual’s right to mental healthcare is in any case indisputable and ratified by the UN, the WHO and the European Union (EU) levels [4]. However, according to a report from the Organisation for Economic Co-operation and Development (OECD) many individuals with mental health problems do not receive necessary treatment, indicating an international treatment gap of approximately 50% depending on the type of mental health problem [2]. In a cross-sectional study conducted in six European countries aimed at estimating the unmet need for mental healthcare at population level, 3.1% of the adult general population reported an unmet need for mental healthcare and 6.5% of those having mental problems were defined as being in need of mental healthcare [6]. Europeans with mental health problems who have received treatment regarded the effectiveness of mental healthcare as low [7]. Concerns regarding the quality of mental healthcare provided in primary care have also been raised by patients who experienced that they were stuck with ineffective medication treatments instead of being provided psychosocial care [8]. This coincides with Barbato and colleagues [9] who state that Europeans seeking help for their mental health problems are often prescribed ineffective treatments. Importantly, if patients experience the mental health treatment as ineffective they are more likely to discontinue their treatment [10], which in turn could jeopardize their recuperation.

**Table 1:** The AAAQ framework [4]

Main concept	Description
Availability	Existence of healthcare facilities, goods and services
Accessibility	Geographical nearness Easy to enter and move in irrespective of functional variation Affordable Provide understandable information
Acceptability	Respectful encounter Non-discriminatory Patient/person in the centre
Quality	Evidence or knowledge-based treatment and services

There are different factors leading to delay in mental healthcare seeking, such as, structural barriers in terms of availability of mental healthcare [10, 11], economic barriers [10, 12], and transportation [12]. Attitudes in terms of a wish to deal with the problem yourself [10] and self-stigma constitute other barriers in seeking professional help for mental problems [10, 12–15]. Rüsch et al. [16] made a distinction between social stigma and the self-stigma that can occur because of the negative attitudes held by other people. Self-stigma has been associated with both less openness to and less perceived value of professional treatment for mental problems [17]. Another phenomenon that has been forwarded as relevant to help-seeking behaviour is mental health literacy [18]. Indeed, Xu and colleagues [19] reviewed the effectiveness of interventions aimed at promoting help-seeking for mental health problems and found that increasing mental health literacy had a positive effect on professional help-seeking.

Important perspectives have emerged from previous qualitative studies. Newman et al. [11] as well as Gilburt et al. [20] have highlighted that the relationship between service users and professionals within mental healthcare is important as it form a basis for interaction and support, which is important to combat mental health problems. A qualitative study on mental health services provided to immigrants in 16 European countries [21] found consistent challenges to ensure optimal treatment for this marginalized group. The authors further suggest that recommendation for best practice may be appropriate at a European level. The majority of previous qualitative studies in this field capturing experiences from people with lived experiences have emerged from single countries, such as the United Kingdom and Ireland. Qualitative studies add to the research field but few studies take a cross-country perspective.

In summary, the burden of mental health problems in Europe challenges healthcare to offer high-standard mental healthcare corresponding to the AAAQ framework but quality improvement should be a continuous effort [4]. So far, most studies and reports of mental healthcare provision in Europe are based on quantitative data. The European Mental Health Action Plan 2013–2020 [3] stipulates that research in mental health assessing needs is to be supported and proposes that persons with mental health problems and their families are involved in quality control. Therefore, conducting qualitative studies that elicit an in-depth exploration of the perspectives of people with lived experiences of mental health problems and the use of mental health services would add to previous research and knowledge by increasing awareness and utilizing the expertise of this group. This can contribute to an in-depth understanding that can inspire and form the basis for quality improvement and development of mental healthcare in Europe.

### Objective

The aim of this study was to describe experiences of mental healthcare among adult Europeans with mental health problems.

## Method

The current study is part of MentALLY - Together for better mental healthcare - a European collaborative effort between Belgium, Cyprus, Greece, the Netherlands, Norway and Sweden. MentALLY is a pilot project that has received funding from the European Parliament and the intention of the project is to gather qualitative data to improve European mental healthcare (http://mentally-project.eu). The current study is based on qualitative focus group interviews.

### Participants

The total number of participants (*N* = 50) were adults recruited for separate focus group interviews in the six countries. All participants had personal experiences of mental health problems and treatment from the mental healthcare services in their countries. The participants’ experiences were varied in type, length and time of contacts with the health care. Table 2 summarizes the recruitment procedures in each country and presents the study population. In all countries, the recruitment was done in several different ways to reach persons that might be interested in sharing their experiences. In all countries the initial contact was followed by written information about the MentALLY project after which the persons made their decision to participate.

**Table 2:** Overview of the focus group interviews and participants

	Belgium	Cyprus	Greece	Netherlands	Norway	Sweden
Number of focus groups	1	2	1	1	1	1
Number of facilitators in each focus group	1	1	2	1	3	2
Length of focus groups in minutes	132	73 + 126	141	123	100	118
Number of participants	14	4 + 4	8	7	6	7
Male participants Age range in years	6 48–63	3 + 2 50–56, 30–32	4 32–55	2 45–69	1 45–55	2 29–48
Female participants Age range in years	8 24–54	1 + 2 45, 50–52	4 32–45	5 44–67	5 40–70	5 27–63
Recruitment through	Previous research database Social media and websites of users’ organisation Mental health care practitioners	Private and public mental healthcare providers	Mental healthcare services and practitioners	Primary and secondary mental healthcare services. Users’ organisations. Websites, newsletters.	Mental health patients’ organisation	User’s organisations University ad in social media
Interviews were held	University facility	One private and one public mental health facility.	Outpatient mental health facility	University hospital	Centrally located outpatient clinic	University facility

### Data collection

Data were collected through focus group interviews between April and November 2018. In total seven focus groups lasting between 73 and 141 min were conducted, each consisting of 4–14 participants (Table 2). MentALLY is a pilot project including six countries to test whether the study design and methods were useful in a cross-country approach. We therefore choose to limit the data collection to one focus group per country with one exception in Cyprus where two focus groups were conducted. The focus groups were facilitated by a social psychologist and a clinical psychologist in Greece, by a social psychologist in Cyprus, by a psychologist in Belgium, by registered nurses in Sweden, by a clinical psychologist, a psychology student and a physiotherapist in Norway and in the Netherlands by a communication scientist supervised by a clinical psychologist. Number of facilitators in each focus groups are accounted for in the Table 2. All focus groups were held in the native language of each country.

A special effort was made to create a pleasant and hospitable atmosphere. Each focus group discussion began by providing information about the MentALLY pilot project and the interview procedures. Permission to audio record the interview was provided by the participants. They also provided written informed consent and demographic information including their age, sex, mental health problems and type of mental healthcare they had experienced. Registration of diagnoses or treatment site was not included in the ethics approval application in Norway, and this information was therefore not gathered at individual level.

The focus group interviews followed an interview guide that was developed in English by the MentALLY teams in collaboration. Thereafter the interview guide was translated into the different native languages using back-translation techniques [22]. The interview guide is presented in Table 3. The interviews started with opening questions regarding positive and negative experiences with mental healthcare to inspire the participants to start talking and thinking about their experiences of mental healthcare. Gradually the questions became more focused relating to the organization of mental healthcare, changes needed to reach the goal of optimal mental healthcare and questions relating to the participants’ experiences of access to mental healthcare, issues related to diagnosis and referral, as well as treatment and collaboration. The interviews ended with questions about uncovered topics and the most important aspects of mental healthcare were discussed [23]. The audiotaped interview material was transcribed verbatim and translated into English in each country. Each participant was given a code to preserve confidentiality.

**Table 3:** List of the interview questions

**OPENING QUESTIONS**	
1. In general, how would you describe well-organized care for people who are confronted with mental health problems?	
2. In general, what are your personal positive and negative experiences with mental health care in your country?	
3. If you think about your own experiences and you could change only one thing to reach the goal of good care for people with mental health related problems in your country, what would that thing be?	
**KEY AND FOCUS QUESTIONS**	
**Access**	
4. What has hindered or helped you in seeking and finding help for your mental health problems?	
5. What would have helped them or would help others in the future in seeking and finding help for mental health problems?	
**Diagnosis and referral**	
6. What are your experiences with receiving the most appropriate help for your specific mental health problems?	
7. What would have helped them or would help others in the future in order to receive the most appropriate help for mental health problems?	
8. What are your experiences with receiving help on time for your specific mental health problems?	
9. What would have helped them or would help others in the future to receive help on time for mental health problems?	
**Treatment**	
10. What are your experiences with the outcome of the treatment(s) you received?	
11. What were, according to you, the specific elements in mental health care leading to recovery from your mental health problems?	
12. What were the elements that hindered you in recovering from your mental health problems?	
13. What would have helped them or would help others in the future to receive more successful treatment for mental health problems?	
**Collaboration**	
14. How would you describe your relationship with the health professionals and services that were involved in your recovery process.	
15. What would have helped them or would help others in the future to come to a better collaboration between the professionals working in mental health care and the people with mental health problems?	
**CLOSING QUESTIONS**	
16. What was the most important issue that we have talked about today?	
17. What topics have we not covered today?	

### Analysis

The initial analysis of the transcripts in the native language was conducted by the MentALLY teams in each country separately, except for the data from Cyprus and the Netherlands that were analysed by the Greek and Belgian teams, respectively. The transcripts and the analysis from each country were then translated into English to enable the creation of a common result covering all countries. The analyses followed the thematic analysis as described by Braun and Clark [24]. Adhering to a common analysis template in all MentALLY teams, the analysis was driven by an analytic interest to acquire a more detailed understanding [24] of experiences of access, diagnosis and referral, treatment and collaboration regarding mental healthcare.

### Ethical considerations

Ethical approvals were granted from the Review Boards at Ghent University in Belgium, the University of Crete in Greece (Επι.Δ.Ε. i.e. Ethical Committee, 6/2018/16-05-2018) and the Regional Ethical Review board in Gothenburg in Sweden (474–18). In Cyprus, the Mental Health Services of the Ministry of Health gave permission to conduct the study (4.2.09.37/7). In the Netherlands, an application was sent to the Medical Ethical Committee of Academic Medical Hospital of the University of Amsterdam and an exemption was given as all subjects were healthy and non-invasive procedures were used (no number was given due to the exemption). The Regional Committees for Medical and Health Research Ethics in Norway deemed the project outside the realm of medical ethics assessment. Therefore, an assessment and approval from data protection officer at the Norwegian Institute of Public Health was collected. Following written and verbal information about the study, all participants signed an informed consent before the focus group interviews started.

## Results

**Fig. 1:**
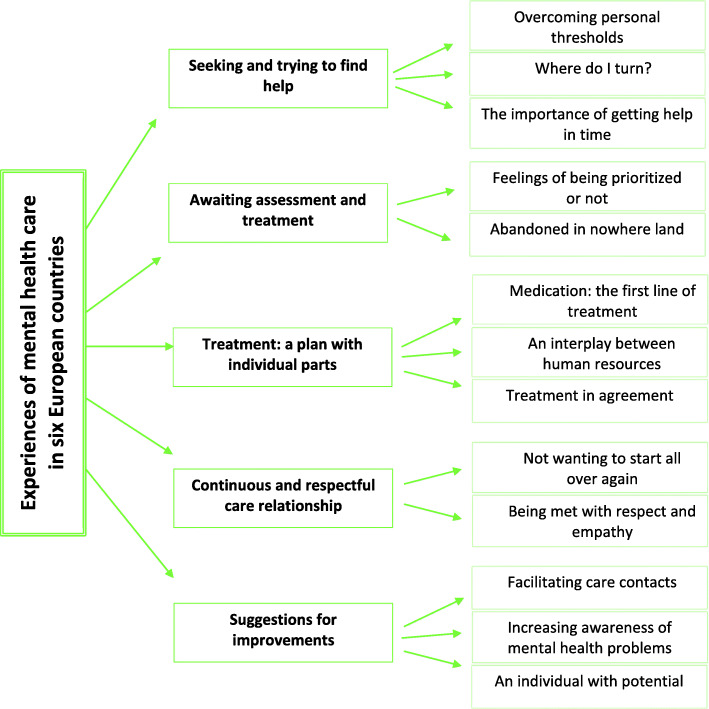
Illustration of themes and subthemes describing experiences of mental healthcare in six European countries

The results are based on the thematic analyses of focus group interviews where a total of 50 participants with lived experiences of mental health problems and of receiving mental healthcare participated. As a group, the focus group participants had experiences from mental healthcare in both private and public sectors, and from in- and outpatient mental healthcare for various mental health problems. The participants had experiences of a wide range of mental healthcare treatment offers. Figure 1 depicts the experiences across five themes and 13 subthemes.

## Seeking and trying to find help

The participants shared their experiences of what hindered or helped them in seeking and trying to find help for their mental health problems. Some of these experiences were related to accessibility to mental healthcare.

### Overcoming personal thresholds

To seek help for mental health problems was described as a process in which the participants had to overcome different personal thresholds. According to some of the participants, one of the first thresholds to overcome was to accept and acknowledge that they were facing mental health problems that required professional care. Furthermore, they needed to be courageous enough to make a decision to contact a healthcare provider, which for some was an easier threshold to overcome than for others.

*“You admit to yourself that something is wrong, and you go to a psychiatrist – the way you go to a GP or any other doctor. Why not? If you have a problem, you need to do that in order to get well”. (3 Cyprus).*

*“There is a threshold for having the courage to contact the care system to begin with.”*

*(4 Sweden)*

*“The first time I came with a very deep depression, it was difficult to seek help, it was shameful.” (4 Norway).*

This process and its thresholds consisted of both internal and external aspects, which could delay or even prevent the participants from seeking mental healthcare. Some were experienced in a context of negative attitudes, stereotypes and other aspects of public stigma surrounding mental health problems.

*“In Cyprus, people with mental health issues are stigmatized” … // … “This is a big problem in Cyprus. Someone may suffer from a mild depression and leave it without treatment because of what society is going to think”.* (1 Cyprus).

*“...// … break through certain taboos, because I believe it is the biggest problem in Belgium. To break through the taboo of psychic health.”* (1 Belgium).

Besides a process of acceptance and courage lived in a context of public stigma, there were other personal thresholds for seeking mental healthcare. For some of the participants these thresholds were related to previous negative experiences with the use of mental healthcare, while others had problems affording the care they needed and consequently were prevented from accessing necessary mental healthcare.

“*… // … this is a very important thing, that you...that there are far too many people who are terrified to have anything to do with mental healthcare, because...and then they have been there, and they know what they are talking about.”* (1 Sweden).

*“Ah yes just financial accessibility for everyone he, because that is the first block for many people because we may have now...// … many people may be lucky enough to have an insurance or I don’t know, but there are also people who have no insurance and mutuality that is refunded … // …* ”(2 Belgium).

*“I couldn’t get an appointment in the public sector. I went to the private sector. … // … There are people that haven’t got the financial resources to get help (in the private sector)”.*

*(4 Greece)*

*“ … // … you don’t have the money to go to a clinic. So what can you do? Where can you go?” (6 Cyprus).*

### Where do I turn?

Related to the above described difficulties uncertainties about where to turn to seek care and treatment i.e. access to mental healthcare were brought up in the focus group discussions. Experiences of not finding the way through “the wilderness” of healthcare offers was mentioned and, as was to not know at all where to turn to receive help. Some found information by chance or by persons in their network. A common experience in the studied countries was the lack of easy found information about how to access mental healthcare.

“*I found it by myself. Circumstances helped me, a higher power if you want. I went to an event, found a pamphlet and I started going there.*” (1 Greece).

*“A friend told me about it – I had no idea such a facility existed.*” (2 Cyprus).

*“Well, I am thinking about when you don’t know exactly where to turn to. Where do I go, what should I do, who should I talk to? It does seem pretty unclear. Where do I begin, where do I call?”* (2 Sweden).

*“I think it is a... a wilderness, that you don’t know where to go.”* (3 Belgium).

*“And plenty exist, but we need to find what is what and what exists,”* (1 Norway).

*“Sometimes unfortunately you get tips that have worked for other, but did not help me. But I do notice that, I have been helped a lot by these tips by others.”* (1 Netherlands).

### The importance of getting help in time

To get help just in time was brought up as an important aspect of access to mental healthcare. Quite some time could pass before contact with a healthcare provider was made. Reasons were associated with the thresholds of acceptance, courage and public stigma that had to be overcome before help was sought, and in addition was the time it took to figure out where to find help. There were also acute situations when urgent contact with mental healthcare was made. Nonetheless, immediate access to mental healthcare was always regarded as crucial. When they contacted a healthcare provider the situation was described as acute or highly open in the sense that the need for help and the wish to get help was combined. This was described as important to combat the mental health problems with support of professionals. However, there were disagreements regarding waiting times as the participants had different experiences of waiting times when contacting mental healthcare. Some had very good experiences of getting help in time and had not been confronted with waiting times.

*“...I haven’t been confronted with waiting times.”* (5 Belgium).

*“I called in the morning, made an appointment and came here (mental healthcare) on the same day. It was all very easy*”. (3 Cyprus).

*“I didn’t have the same experience. My psychiatrist immediately helped me.”* (3 Netherlands).

For others it was more difficult to access mental healthcare due to long waiting times, which appeared to be very common. They described the waiting as being in a desperate situation characterised as a matter of survival or death. Additionally, the long waiting times for treatment resulted in strenuous and unbearable situations for their relatives.

*“Yes, I had to wait several times for a long time. But at the same time, 3 weeks are hellish as well. When you are suicidal for 3 weeks, it becomes too much.”* (1 Netherlands).

“*There are people who have died waiting to be put on the waiting list (to enter a rehabilitation program for their addiction). There are mothers who are really begging out there (in the rehabilitation centre) elderly people praying and crying.*” (3 Greece).

Even as a patient within the psychiatric care system, it was not easy to access the care they needed to deal with their mental health problems. During the experienced long waiting times feelings of resignation emerged. The open window of a need and wish for help seemed to be time dependent.

*“You cannot find the doctors and the professionals when you need them. They are not there when you need them. You need them and you feel they have abandoned you.”* (2 Greece).

*“* … *and when I looked for care through the care system, I didn’t even get an appointment with a psychologist before I gave up and began to self-medicate instead, sort of thing. It is these waiting times that I would emphasize have really been a major obstacle for me.”*

(3 Sweden)

## Awaiting assessment and treatment

Other experiences of the mental healthcare in the focus groups performed in six different European countries were related to how the mental healthcare functioned. When the participants eventually got in contact with healthcare and asked for help to fight their mental health problems, some of them experienced that their health problems were not prioritized and not always carefully assessed while others had experiences of being prioritized and properly cared for. When being referred to another level of care, feelings of being abandoned arose for some participants during the waiting time, because they experienced that no one cared for them. According to these participants, there seemed to be a lack of support in between the appointments and different levels of care. On the contrary, other participants described a smoother referral process to the level of care they deemed correct.

### Feelings of being prioritized or not

Some participants expressed that healthcare professionals did not take mental health problems as seriously as somatic conditions. That experience led in turn to the feeling that healthcare providers did not prioritize mental health problems. In contrast, other participants shared experiences of being prioritized and thus being more or less immediately assessed and referred to correct level of care. Thus, the feelings of being or not being prioritized in care were individual experiences but also understood as structural differences. Some participants felt that they had to exaggerate their problems, and especially the risk of committing suicide, to receive help when being in need. In opposition, other participants shared their positive experiences of how well they had been cared for.

*“I am aware that it isn’t something that is seen with the eye, but still if others can see it, a caregiver should as well. I find that so hard to understand. When it comes to physical problems, they do not just send someone home, they do not say yes, it’s cancer, just rest up a bit and perhaps the tumour goes away.”* (1 Netherlands).

*“I would want it to be easier to get help. Because I have said so many times that I want help, but then I do not get it. And they say to the patients: “have you tried taking your own life”, and “no, then you do not get to be admitted”. But you need to be admitted before you get so sick, in order to avoid getting sick.”* (1 Norway).

*“I think that I have received a lot of good help, actually. I have been treated in the mental health system for many, many, many years, in periods … // … I am still in contact with a psychiatrist for medication and stuff, and I think that I have received a lot of help and support … //...well, mainly through the open mental health system*.” (6 Sweden)

*“I have a few positive stories. For me, I was always referred to a good institute or caregiver. Well not always, but most of the time. And when I was sent to the wrong place, they would send me through to the correct place quite quickly. It never ended up being terrible. Sometimes you hear those stories of how it turns out bad. But it went well, even with files. The referrals in general were good.”* (1 Netherlands).

*“I’ve also been admitted to the psychiatric ward of the general hospital. That was very nice. Everything was ready and waiting for you.”* (4 Cyprus).

Experiences that nurtured the perception of not being a priority were related also to the quality of mental healthcare, and related negative experiences. It was discussed that a careful anamnesis was required to diagnose mental health problems accurately and that preferably a psychiatrist should be involved from the beginning of the diagnostic process. However, the experience of the diagnostic procedure did not always correspond to these requirements. It was argued that time limitations and associated deficiencies in diagnostics and referrals from general practitioners were a major problem, and better support for general practitioners was mentioned as important.

*“When the doctor saw me, he said “you have schizophrenia”. He just looked at me! He did not examine me for more than one minute and he diagnosed me with schizophrenia.”* (1 Greece).

“*… // … that the patient is heard, and seen and gets good help on time, and gets good referrals. The primary doctors need to be strengthened so the patient gets the correct help in the specialist healthcare service that is very, very important.”* (2 Norway).

### Abandoned in nowhere land

The referral process was experienced as long and tiresome with long gaps between inpatient and outpatient care by some of the participants. Without a functional care chain, some experiences concerned feelings of being trapped and abandoned in nowhere land struggling with the mental health problems without support from any healthcare professionals.

*“That it is very difficult, that you must just keep on nagging and fighting, to fight a lot and you don’t have the strength to do that. I find that it is a huge problem, when you are waiting, because you are in the care queue and waiting for a referral, and waiting for something to happen with the referral, and then you just have to keep at them, you have to call, you have to nag, and you don’t have the strength, so you just wait, quiet and being nice. So not much happens. That’s my experience.”* (2 Sweden).

*“It can take a long time before the assessment happens, and in the meantime the patient sits around and is not getting the help he should have.”* (6 Norway).

*“I had experienced it as different islands at the same time. No contact between general practitioner and therapist … // … There was no contact in general between anyone. They all just did their thing, and without me having any say.”* (6 Netherlands).

## Treatment: a plan with different parts

The participants shared their experiences of what aspects in the treatment for their mental health problems that had helped and hindered their recuperation. As previously mentioned, there were thresholds to overcome aggravated by public stigma. Once overcome the time was described as crucial since care was often sought late and when symptoms were severe, and in some cases even life threatening. But also in less severe situations the window between need and motivation to seek help could be closed due to experiences of unfair treatment or feelings of being abandoned. However, the participants also experienced it as a long process to recuperate from the mental health problems. During the discussions in all focus groups, the participants highlighted that it required a combination of different kinds of help and treatment to get better. Depending on individual needs, it seemed important that the treatment was individually planned and that a combination of treatments was the most effective.

### Medication: the first line of treatment

Although the participants argued that an individualized treatment entailing different parts was needed to recuperate, some of them seemed to experience that medication was usually the first line of treatment prescribed. However, there was a variation of experiences regarding the medication treatment. On one hand, some of the participants recognized the importance of medication because either it had improved their condition or because it helped them to avoid negative outcomes i.e. relapses. On the other hand, some of them argued that there was an over-prescription of medication – sometimes against their will.

*“I would be unable to function right now without the medication. I’d be much worse if I didn’t take it. Much worse. The medication I’m taking helps me a lot. If it weren’t for it, I’d take drugs, or start drinking. Taking it is in my best interest – not the doctor’s or the pharmacist’s.”* (4 Cyprus).

“*… // … they press in more and more medicines, and if you try to say, wait a bit, I want some form of therapy or tool so that I can get somewhere with my problem, well then they just prescribe more medication that they don’t follow up and it just gets worse and they don’t check up, sort of thing, whether those medicines work together.”* (2 Sweden).

### An interplay between human resources

In all focus groups, the participants emphasized that medication regimens were not the only solution and therefore they discussed the elements in mental healthcare that they regarded as necessary for them to get better besides medications. Naturally, there was a variation of what elements that was considered as beneficial to get better from the mental health problems. They discussed that different resources were needed to meet their treatment needs and an interplay between resources was seen as treating the patient as a whole person. Among the resources mentioned to be involved in the treatment was healthcare professionals with experiences and competencies from different specializations. Additionally, patients themselves should also play an active part and participate in the treatment planning.

*“In principle, we cannot treat someone with medications without psychological support. I think that everyone must do their part. That is, a person arrives at the doctor, that is, the psychiatrist, he will do his own work, and there must be a psychologist. What does this person have behind him? He has family, children, and legal issues? There should be a social worker who has a different role from the psychologist. It is a different job. … // … There should be different specialties.”* (3 Greece).

*“I think there is much in being able to take part in your own treatment, that you are a part of planning the treatment, and actually writing your own treatment together with your therapist, what sort of treatment you want, what is written in the journal, to be a part of planning the treatment course. However, it will probably be like that now in the set clinical pathways, that you are able to take part in your own treatment.”* (3 Norway).

The continued discussion focused on the benefits and challenges with involvement of significant others in the treatment. Some participants considered significant others as an essential resource. Without them they risked fighting the mental health problems on their own. They argued that their next of kin should be invited to be involved so they could have a better understanding of the situation but also so they could provide the necessary support. In contrast, the involvement of significant others was also experienced as challenging as they could contribute to further distress by being unsupportive. Another challenge, highlighted by some of participants, was to be open about mental health problems and talk with their significant others about their problems.

*“Perhaps professionals could require you bring someone with you during the visit. Things such as a visit from the GP. You first visit a specialist and you forget half of it so that they ask you to make a checklist. Because you are in such a bad place. I have had that problem for my whole life, making a list but never finishing it. Take your mother with you, is how I would describe it. That should become a standard. It is a smart thing to do.”*(5 Netherlands)

*“And it’s also a little hard to hear people tell you that it’s nothing and you’ll get over it. Personally, I had to face that too. My environment didn’t realize I was going through something serious … // … I had to fight on my own in order to make it”.* (3 Cyprus).

“*… // … the word taboo, as long as we don’t break through it and don’t dare to talk with our children and our relatives”* (1 Belgium).

Another element in the treatment that was regarded as essential and helpful to get better was inclusion of people who were experts by experience.

*“Actually the best therapists are those who have experienced it themselves … and sometimes I wonder that is a kind of … if my psychiatrist is sitting in front of me, that is book knowledge … that person can never know what a depression is, that person can never know what an eating disorder is, he knows it but just due to experience, but that person cannot … at first-hand experience it. And the biggest help I got was actually from people with the same problems.”* (1 Belgium).

An important part in the treatment, which facilitated for them to get better was the encouragement of social health i.e., contacts with their social context and labour market. When struggling with mental health problems, it was sometimes difficult to maintain one’s social health. Getting help to maintain or re-establish contacts with the labour market and social networks was considered essential.

*“But it is important to address all problems the person is presenting. It is not certain that the psychiatry or the substance abuse is the worst. It could be economy, it could be living conditions, there are many other things that play a role, it is not only psychiatry”.*(1 Norway)

*“...// … even if the Social Insurance Agency is outside the care system it is an extremely important part of care, because it is the whole reason that you have security in being able to pay your bills and stuff.”* (1 Sweden).

### Treatment in agreement

The importance of consent in different stages of the therapeutic process was discussed during the focus groups. The option to interrupt the therapy and voice concerns towards their care provider was emphasized. Moreover, experiences of involuntary hospitalization and treatment had left some of the participants traumatized and very critical about the practices and the legislation that support these types of interventions. Negative experiences were also related to the reluctance to seek care discussed above.

*“I think that is also still a big problem that a lot of people don’t dare to take a step themselves to … to stop with that... and say, ‘he isn’t doing anything for me, I have to go to somebody else.”* (7 Belgium).

*“This distressed me too much because they took me with handcuffs; they took me in a patrol car as if I was some criminal … // … I have yet to recover from that hospitalization. I have completely compromised; I have fully complied with what the doctor said I did what she told me but deep inside me I am not well to be honest. I’m not well. And this hospitalization was a horrible experience for me. And I have not been able to overcome it and I will never overcome it. They brought me in with handcuffs as though I was a criminal. There should be a court hearing when there is an involuntary hospitalization. No one has called me to defend myself in any court or trial. These things happen only in Greece. This only happens in Greece”.* (1 Greece).

## Continuous and respectful care relationship

When combatting the mental health problems, the participants described that they were in an extremely vulnerable situation. They had overcome the first reluctance to admit their problem and recognized a need to get help as soon as possible. For some of the participants, earlier negative experiences had to be set aside. During this period participants experienced that they were quite dependent on the relationship with the healthcare professionals, who represented the possibility to get better. Thus the characteristics of the care relationship was important to help them recuperate from the mental health problems.

### Not wanting to start all over again

The importance of continuity in care relationships was underlined because that was regarded as helpful in contrast to having to explain everything all over again when meeting new healthcare professionals. Continuity was described as having a steady care contact with one or a few healthcare professionals who were very involved in their care process and who oversaw the whole process and took the necessary steps in the direction of recuperation. The process of needing to start all over again, every time a new treatment started or when having to meet new healthcare professionals, was experienced as exhausting and a hinder to get better.

*“Then just to have a person...yes, partly to one who is...sort of like a coach for that person, a little like a mentor or coach, and that it is one person, so that they are not replaced all the time so you get new people, even if you do not have this first input, it is also important that you have regular staff, ...// …*” (1 Sweden).

*“With my second depression, after my first child, my counsellor, took the initiative, as she saw it was going, left, to come together with me and my general practitioner and my social worker. This instead of having me tell my story over and over again.”* (3 Netherlands).

### Being met with respect and empathy

The situation of being vulnerable was a highlighted experience related to the fact that it was very difficult to stand up for yourself while struggling with mental health problems. However, being met as unique human beings in a respectful manner by healthcare professionals eased the situation. In contrast, stigmatizing attitudes and the often-oppressive way mental health professionals imposed a therapy or a course of intervention was experienced as the opposite: unhelpful and did not promote the collaboration.

*“Everyone should be treated as a person, as a distinct individual as all of us are”* (3 Greece).

*“ I wrote a list before I came here and at the top of the list is respect for the individual and ...yes, I totally agree. I experience strongly that you...just like that you didn’t have a voice any longer, I am used to be listened to and taken seriously. Suddenly, I just felt that, aha, here comes a new mental case.”* (1 Sweden).

*“I wish for better attitudes among healthcare professionals, especially when it comes to the most serious diagnoses. There is a lot of prejudice and stigma, and you often get treated as a diagnosis, and I think that is condemnable.”* (6 Norway).

*“They (the psychiatrists) continue to oppress me. They would tell me you will take it (the medication) or you will go in, or I‘ll go back into the psychiatric hospital”* (1 Greece).

The healthcare professionals’ empathic ability was emphasized as a necessary ingredient in the care relationship. The participants reasoned that empathy made the healthcare professionals able to put themselves in the position of their patients and understand them better, which in turn influenced the quality of care. They wanted to feel a willingness on behalf of the healthcare professionals to help them to get better. A feeling that they were authentically cared for instead of feeling as if they just were a number on a list for the healthcare professional.

*“It means empathy; being able to understand the problem so you can help. It means therapists themselves should be able to feel what each one of us is going through. Because if you don’t, you can’t help”.* (5 Cyprus).

*“After a while I got the feeling I was number five of the day. They would have gaps in knowledge about me and then I would be like ‘Yes, but last time we agreed on this and this.’ I call it empathic ability. They lacked that.”* (7 Netherlands).

*“It is like a conveyor belt, you feel like that when you walk in, so I almost never go to the doctor. But when I go, I have gathered some things and when I get to point three, he does not have more time, and then we have to take it next time, and then it is two months to the next time, if it is not urgent. They have too many patients on their lists.”* (1 Norway).

## Suggestions for improvements

During the discussions of experiences of mental healthcare, the participants came up with different suggestions, which they thought would contribute to quality improvement of mental healthcare and to increase awareness of mental health problems in society.

### Facilitating care contacts

Based on the challenges of getting in contact with the mental healthcare system, the participants discussed several creative suggestions, which were seen as important future possibilities to improve availability and accessibility. One suggestion was an emergency reception like the immediate help available for somatic health problems. This help should be adjusted to the kind of mental health problems the patients are struggling with and be based on a correct diagnosis. Besides an emergency reception, the participants also suggested care contact in the form of a knowledgeable contact point that could navigate the care seeker to the correct mental healthcare. Additionally, the availability of an informative website for finding help was regarded as good practice.

*“It would like to have a kind of basic post for mental healthcare. Kind of like an EHBO (First aid), where you could go for help. It would help lots of people. There would be a faster process, instead of the long waiting lists. It would cost the taxpayer less as well.”* (1 Netherlands).

*“I was thinking that if there was...well, you have Contact Point in mental health, if that could have a double function so that you call there, explain that you are feeling ill with this and that, but I don’t know where to turn. If they had the knowledge to then direct you onwards, like railway points in some way. Not just that you can book appointments there, but also that you call there and sort of...I don’t know, where do I go now, and they can help you and direct you onwards, that could be a good thing to have, if you could just talk on the phone.” (2 Sweden).*

Another suggestion was that healthcare professionals should send patients in the right direction by explaining different types of available treatments for their specific problems and the purpose of these treatments. It could be described as an expectation that professionals work not only for their own type of treatment but is aware of and have knowledge in other type of treatments and willing to refer patients further.

*“There are infinite psychologists but if you are no psychologist you don’t know... // … cognitive behaviour therapy what does that include, you have a psychologist that works in a certain manner...but we are not aware of it and I believe that they should explain to us everything that exists”.* (5 Belgium).

“*… // … I also think about this thing about...there has been a lot of focus on CBT, cognitive behavioural therapy, now, and it...I have received another type of therapy, and also have had CBT, and I feel it like this, that CBT is not always suitable, just that, it is very narrowly focused. A lot of people need a completely different type of therapy, but it is just that everybody needs to fit in the same folder today. And I don’t think that it is good. It must be changed.”* (6 Sweden).

Other resources that would presumably improve accessibility to mental healthcare are having a better geographical spread of mental healthcare services or an expansion of mobile teams providing home consultations on demand.

*“I think that, instead of having a bigger building, the facility should spread to other cities … // … We need to have such facilities close to us. It’s not enough for me to come here and see a doctor for 10 or 30 minutes. Or to have to wait for 3 months, for 2 months at least or for 1 month until the date of my appointment comes*”. (2 Cyprus).

*“And then there are these mobile teams, … //... if you have established a contact they can come on home visits, and that is...// … and that is exactly a step that I think would help very many people,”* (4 Sweden).

### Increasing awareness of mental health problems

The participants discussed their wishes for more proactive efforts in society aimed at reducing stigma and increasing knowledge about mental health. Suitable arenas for such initiatives were for example schools and workplaces. It was suggested that mental health promotion should start already in primary schools as a way of increasing awareness and understanding about mental health problems.

*“So, I think there should be an education campaign. … // … we need to get to young people in schools. So that young people know what can happen to anyone. Because what’s happening to me may happen tomorrow to my neighbour. They should know how to handle it, know how to deal with it. Now there are drugs, treatments, and institutions. There are many good and remarkable doctors. So, I think people need to be informed”.* (3 Greece).

*“One thing is that, I think that we touched on it earlier, but that you could also work quite a lot proactively on public health and have more healthy living thoughts that have more focus on the mental health parts, too, that you can hear about it in school at an early age, and I think that it would be an incredibly important tool for public health. As we said earlier, building up this knowledge, too, that you can sort of...I don’t want to say normalise, because I think that would give a negative connotation, but in some way still normalise mental illness.”*(3 Sweden)

Some participants described the usefulness of experts with experience being involved in team meetings, in meetings with politicians, in education for healthcare professionals. They described that involving former patients might broaden up new perspectives above and beyond theoretical knowledge. By being involved in these things, they wanted care providers and policy makers to understand what mental health problems are really like.

*“I think that it’s very important that there is uhm good education but there is still a big difference between book knowledge and experience knowledge and if both sectors would cooperate I think that...//... that there would be more understanding, much more indeed... as (name participant) said about changing that mentality or saying ‘politics’ or so, that doesn’t go … doesn’t go so easily but we, from our difficult things we can convert it in that positive one and work with that, witness, to inform people correctly.”* (9 Belgium).

### An individual with potential

The focus group discussions had a clear message that it was important to be treated as individuals with potential, with strengths in addition to vulnerabilities and with ability to strengthen their health. This was something that the participants meant could improve treatment for mental health problems. Some participants also suggested that alternative treatment options which they meant could facilitate for them to recuperate from the mental health problems were included in the treatment. Importantly, the treatment should not focus solely on the mental health problems but also on the healthy parts that could be strengthened.

*“It is important that the care providers look at what our talents are, our powerful points and not just at what is going wrong.”* (8 Belgium).

*“ … // … to treat an illness is also to...you need to sort of treat the people who are ill … // … strengthening the healthy, because focusing too much on the illness makes you more ill, and then it is easy to be, sort of...it goes in a ring, a downward spiral. And exactly that somewhere in care...exactly that thing about the individual, empowered, not just focusing on the illness but also ensuring that you do things that strengthen and retain function. I think, that is how it works in the somatic world, strengthening and retaining, and that should also exist in mental healthcare, I think. And that should be of political interest, too.”* (4 Sweden).

## Discussion

The aim of this study was to describe experiences of mental healthcare among adult Europeans with mental health problems. Focus groups were chosen as a suitable form of data collection allowing the participants to share and discuss their experiences of mental healthcare. Experiences of seeking and trying to find help were related to overcoming personal threshold of accepting the problems and need for care, not knowing where to turn and to get help in time. Awaiting assessment and treatment included feelings of being prioritized or not and feelings of being abandoned. Experiences of treatment disclosed a need for more tailored treatment plans including more pieces than medication treatment. Continuity in mental healthcare and being met with respect and empathy were raised as crucial in care relationships. Possibilities for future improvements were put forward entailing suggestions to increase the societal awareness of mental health problems.

The AAAQ framework refers to availability, accessibility, acceptability and quality in healthcare [4]. Interestingly, all aspects of this framework came up in different ways in the focus group interviews. This was of particular interest since the study was not designed with AAAQ in mind, it did not guide the focus group interviews and it was not used as an analytical tool during the analysis of the interviews. However, the AAAQ were used as a tool to discuss the findings and mirror the users’ experiences in a framework based in rights and expert opinions. Availability was the least discussed aspect, but several suggestions of mental healthcare facilities that were non-existing were mentioned such as a special first aid emergency unit for persons with common mental health problems, since fast and good quality support was seen as helpful in general. It was considered a way to secure the emotional ground and as a help to get well. Mental health first aid programmes introduced by Kitchner and Jorm [25] have been evaluated as effective for provision of first aid support for individuals developing mental health problems [26]. This started as an effort to educate lay persons to recognize and provide a first support for someone with mental health problems prior to a professional care contact being established [25]. Mental health first aid can be a form of available help for individuals developing mental health problems, but it needs to be aligned with available professional mental healthcare that is easily accessible.

Experiences of accessing mental healthcare were clearly addressed during the focus group interviews. Accessibility refers mainly to the idea that healthcare should be easy to access, affordable and geographically close [4]. The current results showed that some participants experienced structural barriers in terms of difficulties in accessing mental healthcare but also barriers related to personal economic conditions, which is in accordance with previous research highlighting insufficient accessibility [11] as obstructing mental healthcare seeking. Geographical closeness of mental healthcare facilities was seen as important by the participants and they gave creative suggestions to improve access. One suggestion was mobile teams that could do home visits on demand. Mobile mental health units have been proven successful in remote areas in Greece by reducing hospitalizations. These units also seem to encourage patients with psychosis to receive treatment thanks to accessibility and non-restrictive care [27]. A similar French initiative in terms of a mobile mental intensive care unit has been evaluated as functional for individuals with their first episode of psychosis and those being at risk of mental health problems [28]. Another suggestion was to build smaller units throughout a region instead of large hospitals in urban areas. Thus, moving mental healthcare closer to those in need instead of centralizing might be one option to increase both availability and accessibility.

Accessibility also stipulates that information about healthcare should be provided in an understandable way [4] but importantly, the participants displayed a need for information about mental healthcare. An interesting angle to this was brought up by the focus groups participants who wanted a broader information on accessible treatment methods. They had experienced that professionals often were devoted to a specific type of care (cognitive behaviour therapy or medication to mention two) while participants wanted a broader palette to choose from. This is particularly interesting in the ongoing development of evidence based healthcare; certain types of treatments receive less attention or are more difficult to investigate which give them a disadvantage on the healthcare market, be it private or public.

Furthermore, the experiences regarding accessibility also contained expressions of uncertainty about where to turn when having overcome the threshold to actually seek mental healthcare. This expressed need for information about how to access mental healthcare and different treatment methods could be understood as a need for better mental health literacy. Mental health literacy has been described as relevant for seeking mental healthcare [18] and interventions aimed at increasing mental health literacy have shown a favourable effect on seeking mental healthcare [19]. A suggestion from the focus group participants was that web-based tools could be used to improve access information. Web-based interventions targeting mental health literacy have been proven effective if they contain a structured program, are directed towards a specific population, provide evidence-based content in a pedagogical manner and are interactive [29].

The current results also demonstrated a wish for more proactive efforts in society aimed at reducing stigma and increasing general awareness of mental health problems in society, which could be interpreted as a plea for better general mental health literacy. Information campaigns in schools were suggested, which is in line with previous research showing that efforts to improve mental health literacy in schools are effective among adolescents. Campaigns both in the community and in schools can improve mental health literacy in general leading to improved readiness to act on mental health problems [30, 31]. It could be argued that the current results point towards a clear harmony between experiential knowledge from the perspective of people with lived experiences and scientific evidence, but there is a gap without a firm bridge connecting these knowledge practices. One scope of the European mental health action plan is to establish accessible care that meets peoples’ needs [4]. Hence, initiatives focusing on improving mental health literacy may be one notion for policymakers to consider. This corresponds to both patients’ experiences and scientific evidence as well as the AAAQ framework which emphasizes the importance of evidence-based knowledge regarding treatment, and service as a quality criterion [4]. In fact, it can be hypothesized that an improved population mental health literacy in the population will reduce stigma and improve individual mental health literacy. Policymakers, mental healthcare and healthcare professionals are obviously important drivers in such a process.

It was obvious that there were thresholds to overcome before seeking mental healthcare. The first was to acknowledge that one had mental health problems and then to ignore stigmatizing attitudes. These experiences are in accordance with the results in a review by Newman et al. [11] showing that seeking mental healthcare is a complex effort involving aspects such as overcoming stigma but also prolonged suffering among individuals who did not admit that they needed help. Regarding stigma, reviews show that stigma is associated with mental healthcare seeking [13–15]. It was evident that stigma prevailed in all countries included in the present study both as social- and self-stigma and perhaps of outmost importance stigmatizing attitudes from healthcare professionals. Because stigma appears to influence mental healthcare seeking [12–14] and as a result most likely delay initiation of treatment or perhaps even worsen the mental health condition, actions are urgent. Unmet needs for mental healthcare can lead to secondary negative consequences such as reduced capacity to work and sickness absence. Essentially, our study also showed that third parties i.e. next of kin are affected by worries, responsibilities and reduced quality of life. Before Europe can move towards a better mental healthcare, stigmatizing attitudes need to be combatted on all levels in society.

From the experiences shared in the focus groups, it was clear that individualized care was requested containing different parts tailored to each patient’s problems and needs. Being treated as a routine patient was not supportive in the process of combatting the mental health problems according to the participants in the current study. In fact, person-centred care with healthcare professionals working in teams involving patients and their families has been put forward as a future direction of mental healthcare [32], which is also in line with acceptability in the AAAQ framework underlining placing the patient/person in the centre [4]. Among the needs that focus group participants mentioned were social treatment including support to become knowledgeable of how society works and being prepared to make contacts with authorities. Participants mentioned the vulnerable situation a person with mental health problems encounters and that this vulnerability can be a major barrier. Mental health problems impact on ability to participate in the labour market [2] and may even postpone return to work [33]. It is therefore of vital importance to remember social health when planning treatment for persons with mental health problems. An enhanced collaboration between mental healthcare, social authorities and policymakers is most likely an urgent matter to develop strategies aimed at supporting individuals with mental health problems to improve their social health and to act on exclusion from the labour market and society in general. In this respect, empowerment could play an important role as previous research has demonstrated associations between empowerment and occupational engagement among persons with mental health problems [34]. It was brought up during the focus group discussions that is was important to be seen as an individual with potential, which can be interpreted as a wish to be empowered. The WHO action plan [3] has also emphasized that empowerment is one important value to defend among persons with mental health problems. It has also been argued by McAllistar and colleagues [35] that empowerment serves as an essential outcome measure for patients with long-term health conditions. Because empowerment is associated with both well-being and rehabilitation among persons with mental health problems [36, 37] efforts to enhance empowerment is suggested as one crucial part of mental health treatment.

Another aspect of acceptability is the provision of a respectful encounter [4] which may be related to the results illustrating the importance of continuity and a desire to be met in a respectful empathic way by the healthcare professionals. Continuity has been discussed in many healthcare contexts and both patients and healthcare professionals ask for better continuity [38]. Again, patients with mental health problems might be more vulnerable to a lack of continuity since trust, confidence and treatment alliances between the healthcare professional and the patient are fundamental ingredients in mental healthcare. Continuity in mental healthcare is a prerequisite in order to build care relationships [11] and having a trusting care relationship with one or a few healthcare professionals has been considered as good continuity because it means personal stories do not have to be recounted every time [39]. This is in line with the current results showing that the participants asked for better continuity because they did not want to start all over again when contacting mental healthcare. Moreover, being met in an empathic respectful way was also essential because struggling with mental health problems was experienced as being in a very vulnerable situation, often without the resources to make one’s voice heard. Moreover, negative experiences from former care contacts seemed to nurture a negative expectation spiral leading to delays in taking new contacts when their mental health worsened. This delay may lead to impaired prognosis and poorer opportunity to recuperate and in turn to potential higher costs for society in terms of sick leave and healthcare. Thus, a respectful and empathic encounter was called for which has also been found in previous research with persons who have experienced mental health problems [40]. It seems important to develop competence in healthcare professionals to manage their own negative attitudes to mental illness and promote supportive behaviour. Therefore, the current study strongly suggests that syllabuses in educations leading to professions within healthcare and social services contain learning activities focusing on patient/person centred care and empathic and respectful communication and encounters.

Quality in healthcare, which refers to evidence or knowledge-based treatment and services [4], was not explicitly framed in the experiences in the current study, but aspects of quality were embedded in the discussions, for instance in relation to the treatment. In accordance with previous research [8], the participants raised concerns regarding treatment. They experienced that medication was the first line of treatment prescribed and that over-prescriptions occurred. Although they acknowledged the value of medications, they asked for a treatment entailing different parts and not medication alone. Cuijpers et al. [41], showed that combining pharmacotherapy with psychotherapy in adults with common mental health problems appears to be more effective than pharmacotherapy alone. Another suggested part of the treatment that could be related to quality was to involve significant others in terms of persons with experiences of mental health problems and next of kin. Persons with their own experiences, so called peers, were regarded as trustworthy because they shared similar experiences as the participants. In fact, peers can help to combat stigma and being part of a peer support network can offer space to focus on other aspects than mental illness [42]. Concerning involvement of next of kin, it has been suggested that families are invited to be involved in the treatment [43]. Family involvement has for instance resulted in reduced relapses and hospitalizations for individuals with schizophrenia [44]. Cohen et al. [45] found that many individuals with mental health problems wanted their families to be involved but that it could interfere with privacy and integrity and therefore needed to be individually negotiated. The participants in the current study displayed an openness to involve next of kin because thanks to their support they avoided fighting the mental health problems on their own. Furthermore, involving next of kin when receiving information from healthcare professionals was regarded as a quality check because the information was sometimes difficult to remember and understand. In light of the fact that approximately 84 million Europeans have mental health problems [2], even more are affected as next of kin, which may point towards a shift from patient/person centred to family focused mental healthcare comprising treatment with different parts individually determined. Thoughts about a more multifaceted treatment, which was raised in the focus group discussions, can of course be seen in relation to available resources in the society and within mental healthcare. However, a treatment that does not lead to improved mental health is not profitable neither from an individual perspective nor from a societal perspective. Based on the current study, there is a need for more flexible and individualized treatment for persons with mental health problems. Of course, this must be seen in relation to realistic resources but also in relation to costs of treatment failure. As a suggestion, more intervention studies are needed to evaluate the effect of more flexible or person-centred mental health treatments with individual recuperation and cost effectiveness as outcome measures.

### Methodological considerations

A strength of the current study is that the participants represent several European countries and that there is a variation in age, mental health problems and experiences from different forms of mental healthcare. Another strength is that different ways of recruiting were used to ensure a good spread in patient experiences. For example, if participants had only been recruited from psychiatric specialist clinics the experiences would probably have been less varied. Another aspect regarding the recruitment is that all focus group interviews were conducted in urban areas which may indicate that most experiences are related to mental healthcare in larger cities. However, no information about geographical location of the received mental healthcare was collected, which could be regarded as a short-coming and interfere with the applicability of the results. Nevertheless, future studies should preferably address experiences of mental healthcare outside larger cities. As previously described, MentALLY is a pilot project including six countries to test whether the study design and methods were useful in a cross-country approach, which is to be considered as a strength. But a limitation is that only one focus group was performed in each country, except from in Cyprus where two were conducted. As in all qualitative research it is important that the results are not interpreted as possible to generalize in a similar sense as for quantitative studies. The transferability of the findings would have been strengthened with more focus groups in each country not related to mere numbers but rather to a broader patterns of experiences represented in the groups. Comparisons between countries have been avoided.

Moreover, because the participants were recruited through a self-selection process, the findings might not reflect the broader population of people with lived experiences of mental healthcare. To ensure trustworthiness of the results, the analysis was first conducted in each country in the native languages in order to reduce the risk of misinterpretations. The analysis was also supported with verbatim quotations, which could be considered as a strength. As the focus group interviews were held in the native languages, nuances might have been lost in the translation process, an inherent challenge in studies relying on different languages. Regarding trustworthiness, during the analysis, preliminary findings have been presented and discussed with stakeholders from non-governmental network organizations connected to the MentALLY pilot project. Another potential limitation may be that the Dutch data were analysed by the MentALLY team in Belgium but the team in the Netherlands scrutinized the content of the results. It is to be noted that all teams have read the results and contributed with intellectual and critical input. The focus group interviews were conducted adhering to a strict study protocol including an interview guide for conformity between the six MentALLY teams. The interview guide was not pilot tested, which could be regarded as a shortcoming. This and the fact that the analysis followed an analysis template and was driven by what Braun and Clark [24] refer to as an analytic interest to acquire a more detailed analysis of preconceived aspects could have resulted in aspects of experiences of mental healthcare not being covered in this study. Still, the intention with qualitative research is to gain an in-depth understanding of a phenomenon in this case mental healthcare with focus on access, diagnosis and referral, treatment and collaboration in six European countries from a patient perspective and this intention is regarded as reached.

The present study is limited as it involved a small sample of people with lived experiences from each of the involved countries. However, qualitative studies as the present are not designed for wide generalizability, but to gain a rich and deeper understanding of variations of human experiences [46], and our results should be appraised accordingly. As this study is a pilot project to explore experiences of mental healthcare across countries and potentially learn from these, detailed analyses linking their experiences to the specific healthcare infrastructures in each country were beyond the scope of the current study. However, based on the current study, we suggest at least three methodological adjustments would be beneficial: First, we would suggest a mixed method approach comparing experiences from patients to a detailed overview of the treatment modalities and capacity in mental health care. Second, we would recommend an immersed qualitative study to explore questions and findings related to stigma further. This includes stigma from health care personnel, which the participants reported across all focus groups. Third, we would aim to also include survey data to approach a wider sense of generalizations and comparisons of key findings between countries. The findings from the current pilot project has been important to illustrate that a cross-country comparison study with a qualitative approach is feasible. The results indicate that there are similar and cross-cutting experiences and questions in mental health care that should be addressed for several European countries.

## Conclusion

The following suggestions emerged: Mental healthcare can become more accessible through mobile teams and e-health, a steady contact can facilitate the process of diagnosis and referral, mental health treatment needs to be tailored for each individual containing more parts than medication but with the addition of an empathic respectful encounter. Increased collaboration between stakeholders to act on stigma and improvement of mental health literacy is warranted.

## Data Availability

The data collected and analysed during the current study are not publicly available due to research ethical reasons.

## Abbreviations

AAAQ: Availability, accessibility, acceptability and quality of healthcare
EU: European union
OECD: Organisation for economic co-operation and development
UN: United Nations
WHO: World health organization
